# Epigenome‐wide analysis of frailty: Results from two European twin cohorts

**DOI:** 10.1111/acel.14135

**Published:** 2024-02-27

**Authors:** Jonathan K. L. Mak, Asmus Cosmos Skovgaard, Marianne Nygaard, Laura Kananen, Chandra A. Reynolds, Yunzhang Wang, Ralf Kuja‐Halkola, Ida K. Karlsson, Nancy L. Pedersen, Sara Hägg, Mette Soerensen, Juulia Jylhävä

**Affiliations:** ^1^ Department of Medical Epidemiology and Biostatistics Karolinska Institutet Stockholm Sweden; ^2^ Department of Pharmacology and Pharmacy, Li Ka Shing Faculty of Medicine The University of Hong Kong Hong Kong China; ^3^ The Danish Twin Registry and Epidemiology, Biostatistics and Biodemography, Department of Public Health University of Southern Denmark Odense M Denmark; ^4^ Faculty of Social Sciences (Health Sciences) and Gerontology Research Center (GEREC) University of Tampere Tampere Finland; ^5^ Institute for Behavioral Genetics, University of Colorado Boulder Colorado USA; ^6^ Department of Psychology University of California Riverside California USA; ^7^ Department of Clinical Sciences, Danderyd Hospital Karolinska Institutet Stockholm Sweden; ^8^ Department of Clinical Genetics Odense University Hospital Odense C Denmark

**Keywords:** aging, DNA methylation, epigenetics, frailty

## Abstract

Epigenetics plays an important role in the aging process, but it is unclear whether epigenetic factors also influence frailty, an age‐related state of physiological decline. In this study, we performed a meta‐analysis of epigenome‐wide association studies in four samples drawn from the Swedish Adoption/Twin Study of Aging (SATSA) and the Longitudinal Study of Aging Danish Twins (LSADT) to explore the association between DNA methylation and frailty. Frailty was defined using the frailty index (FI), and DNA methylation levels were measured in whole blood using Illumina's Infinium HumanMethylation450K and MethylationEPIC arrays. In the meta‐analysis consisting of a total of 829 participants, we identified 589 CpG sites that were statistically significantly associated with either the continuous or categorical FI (false discovery rate <0.05). Many of these CpGs have previously been associated with age and age‐related diseases. The identified sites were also largely directionally consistent in a longitudinal analysis using mixed‐effects models in SATSA, where the participants were followed up to a maximum of 20 years. Moreover, we identified three differentially methylated regions within the *MGRN1*, *MIR596*, and *TAPBP* genes that have been linked to neuronal aging, tumor growth, and immune functions. Furthermore, our meta‐analysis results replicated 34 of the 77 previously reported frailty‐associated CpGs at *p* < 0.05. In conclusion, our findings demonstrate robust associations between frailty and DNA methylation levels in 589 novel CpGs, previously unidentified for frailty, and strengthen the role of neuronal/brain pathways in frailty.

## Graphical abstract

To assess the association between DNA methylation and frailty, this study presents a meta‐analysis of epigenome‐wide association studies (EWASs) of the frailty index in 829 individuals from the Swedish Adoption/Twin Study of Aging (SATSA) and the Longitudinal Study of Aging Danish Twins (LSADT). A total of 589 CpG sites and 3 differentially methylated regions (DMRs) were statistically significantly associated with frailty. These findings suggest that pathways common to neuronal aging and tumor growth may play important roles in frailty.

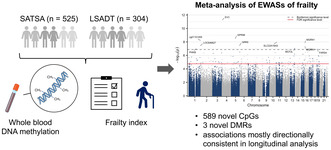

## 1 INTRODUCTION

DNA methylation is a dynamic epigenetic mechanism that can affect regulation of gene expression through the addition of methyl groups to specific locations in the DNA, typically at cytosine‐phosphate‐guanine (CpG) sites (Moore et al., 2013). Methylation levels at these sites are regulated both by genetic and environmental factors (Moore et al., 2013). DNA methylation patterns change with advancing age (López‐Otín et al., 2023) and are associated with various age‐related diseases, such as cancer and Alzheimer's disease (Darwiche, 2020; Mc Auley, 2021; Tecalco‐Cruz et al., 2020). Frailty is an age‐related, complex and multifactorial condition characterized by an increased vulnerability to stressors due to decline in multiple physiological systems (Hoogendijk et al., 2019). Frailty is influenced by the interplay of numerous genetic and environmental factors throughout life (Livshits, Ni Lochlainn, et al., 2018; Mak et al., 2023) and could also be influenced by epigenetic factors. Deciphering the link between DNA methylation and frailty can aid in identifying novel biomarkers for the etiology of frailty and understanding the underlying biological mechanisms of frailty.

To date, only a few studies have examined DNA methylation in relation to frailty. A previous cross‐sectional study observed a significant relationship between frailty and promoter‐specific CpG island methylation, but not genome‐wide methylation, in oldest‐old adults (Collerton et al., 2014). Another longitudinal study found a significant association between a worsening frailty status and decreased global DNA methylation level over a 7‐year follow‐up period (Bellizzi et al., 2012). Other epigenome‐wide association studies (EWASs) of frailty, which are mostly cross‐sectional in nature, have also identified several individual frailty‐related CpG sites (Gale et al., 2018; Kim et al., 2018; Li et al., 2022; Livshits, Malkin, et al., 2018). Nevertheless, no CpG site has thus far been consistently associated with frailty across different populations. The scarcity of longitudinal studies assessing whether CpG methylation levels are associated with changes in frailty over age has also limited our understanding of individual differences in frailty trajectories and differential DNA methylation.

In this study, we performed a meta‐analysis of EWASs on frailty in four samples of Swedish and Danish twins comprising a total of 829 individuals to explore the association between frailty and whole blood DNA methylation at CpG sites across the genome. The identified frailty‐associated sites were also examined for their longitudinal associations with frailty over age up to 20 years in the Swedish cohort. We also provide heritability estimates for the frailty‐associated sites.

## 2 METHODS

### 2.1 Study population

This analysis included 829 participants drawn from four samples of the Swedish Adoption/Twin Study of Aging (SATSA) (Finkel & Pedersen, 2004) and the Longitudinal Study of Aging Danish Twins (LSADT) (Pedersen et al., 2019): (1) SATSA 450K (*n* = 379), (2) SATSA EPIC (*n* = 146), (3) LSADT 1997 (*n* = 304), and (4) LSADT 2007 (*n* = 86) all of whom had participated in LSADT 1997.

Swedish Adoption/Twin Study of Aging is a longitudinal, population‐based study drawn from the Swedish Twin Registry and consists of same‐sex twin pairs over the age of 50 (Lichtenstein et al., 2006). It includes 10 in‐person testing (IPT) waves performed in approximately 3‐year intervals between 1984 and 2014, during which the participants completed a comprehensive health examination, responded to questionnaires, and provided blood samples. Frailty and whole blood DNA methylation data were available in six waves from the third (1992–1994), fifth (1999–2001), sixth (2002–2004), eighth (2008–2010), ninth (2010–2012), and tenth (2012–2014) IPT waves. DNA methylation in SATSA was measured by either the Illumina's Infinium HumanMethylation450K or MethylationEPIC array. To minimize batch effect due to array type, we performed EWAS analyses separately in 379 and 146 SATSA participants with DNA methylation measured using the 450K and EPIC arrays at baseline, respectively (referred to as the “SATSA 450K” and “SATSA EPIC” samples hereafter). Since not all individuals participated in every IPT, we considered the first available measurement as the baseline for each participant. The number of included SATSA participants across waves is as follows: IPT3 (450K: *n* = 230; EPIC: *n* = 76), IPT5 (450K: *n* = 106; EPIC: *n* = 57), IPT6 (450K: *n* = 26; EPIC: *n* = 6), IPT8 (450K: *n* = 14; EPIC: *n* = 7), and IPT9 (450K: *n* = 3).

Longitudinal Study of Aging Danish Twins is part of the Danish Twin Registry and consists of 70+ year‐old twins from same‐sex twin pairs with questionnaire and examination data collected biennially from 1995 to 2007 and whole blood samples collected in 1997 and 2007 (Pedersen et al., 2019). We performed EWASs in both the baseline cohort of 304 LSADT participants (“LSADT 1997”) and the follow‐up cohort of 86 LSADT participants (“LSADT 2007”) who had data on both frailty and whole blood DNA methylation.

All participants from SATSA and LSADT have given informed consent to participate. SATSA was approved by the Regional Ethics Review Board in Stockholm, and LSADT was approved by the Regional Committees on Health Research Ethics for Southern Denmark.

### 2.2 Frailty

Frailty was measured using the frailty index (FI), which operationalizes frailty as the accumulation of age‐related health deficits including diseases, signs, symptoms, and physical functioning (Searle et al., 2008). A 42‐item FI has previously been developed and validated in the SATSA cohort (Table S1) (Jiang et al., 2017; Raymond et al., 2020). It was calculated according to the standard procedure, i.e., the sum of deficits divided by the total number of items considered (Searle et al., 2008), and the included deficit items in each wave of SATSA were identical. Similarly, a 43‐item FI and a 36‐item FI were constructed in the LSADT 1997 and LSADT 2007 cohorts using the same procedures, respectively (Table S1). The FI was used as a continuous variable in the main analysis. Additionally, we considered the FI as a categorical variable using previously described cut‐offs (non‐frail: FI ≤0.1; prefrail: 0.1< FI ≤0.21; frail: FI >0.21) (Rockwood et al., 2011).

### 2.3 DNA methylation profiling

In SATSA, DNA extracted from whole blood was bisulfite‐converted and hybridized onto either the Infinium HumanMethylation450K or MethylationEPIC array (Illumina, San Diego Inc., CA, USA). Details of the blood sample collection and preprocessing of the methylation data in SATSA have been described elsewhere (Karlsson et al., 2021; Wang et al., 2018). Briefly, samples were excluded if they had poor correlation with genetic controls that target single nucleotide polymorphisms instead of CpGs (as an indication of sample contamination) or had a wrongly predicted sex based on the signal ratio from sex chromosomes. Probes were excluded if they overlapped with a single nucleotide polymorphism, had a detection *p*‐value > 0.05, or resided on sex chromosomes. Methylation data were normalized using the “dasen” method from the *wateRmelon* R package (Pidsley et al., 2013), corrected for batch effects using the “ComBat” method from the *sva* R package (Leek et al., 2012), and adjusted for cellular compositions using the Houseman method (Houseman et al., 2012) based on a blood cell reference panel (Reinius et al., 2012).

In LSADT 1997 and 2007, DNA methylation was measured using the Infinium HumanMethylation450K array, and similar preprocessing steps as in SATSA were performed as detailed previously (Soerensen et al., 2019, 2022).

### 2.4 Statistical analysis

#### 2.4.1 Cohort‐specific EWAS analyses

We first conducted four cross‐sectional EWASs of frailty in SATSA 450K, SATSA EPIC, LSADT 1997, and LSADT 2007 samples using generalized estimating equations where DNA methylation levels of CpG sites (i.e., normalized *β* values, representing proportion of methylation) were used as the dependent variables and FI as the independent variable. The models were adjusted for age, sex, smoking, and body mass index (BMI). Both twins from each pair were included in the models, and we accounted for twin relatedness using cluster‐robust standard errors. As the methylation data in LSADT had not been corrected for batch effects and cellular compositions during preprocessing, we additionally included batch and estimated cellular compositions (CD4 cells, CD8 cells, natural killer cells, monocytes, granulocytes, and B cells) (Houseman et al., 2012) in the LSADT EWAS models to enhance comparability of the results across cohorts. We considered the FI both as a continuous variable (per 0.1 increase) and a categorical variable (frail vs. non‐frail and prefrail vs. non‐frail).

#### 2.4.2 EWAS meta‐analysis

A fixed‐effect inverse variance weighted meta‐analysis was performed to combine results from the four EWASs in SATSA 450K, SATSA EPIC, LSADT 1997, and LSADT 2007 samples using the *Metafor* R package (Viechtbauer, 2010). The meta‐analysis included 368,249 CpGs that were available in the three 450K samples (i.e., SATSA 450K, LSADT 1997, and LSADT 2007). Potential heterogeneity was assessed using the *I* ^2^ value (Higgins et al., 2003). CpGs associated with either the continuous or categorical FI at a false discovery rate (FDR) <0.05 were considered statistically significant (Benjamini & Hochberg, 1995).

#### 2.4.3 Functional enrichment

We performed gene ontology (GO) and Kyoto Encyclopaedia of Genes and Genomes (KEGG) pathway analyses using the *gometh* function of the *missMethyl* R package, which performs a hypergeometric test that accounts for the different number of CpGs per gene (Phipson et al., 2016). The CpGs identified from the EWAS meta‐analysis were taken as the input, and the 368,249 investigated CpGs were used as the background set of CpGs. We accounted for multiple testing using the FDR adjustment.

#### 2.4.4 Differentially methylated region analysis

In addition to the EWAS meta‐analysis of the individual CpG sites, we performed differentially methylated region (DMR) analyses in the four samples and meta‐analyzed the results using the *dmrff* R package, a method that combines summary statistics of nearby CpGs and has been shown to have high statistical power to detect DMRs (Lent et al., 2021; Suderman et al., 2018). A Bonferroni‐adjusted *p* < 0.05 was considered statistically significant in the DMR analysis.

#### 2.4.5 Look‐ups of the findings

We looked up the previously reported traits associated with the CpGs identified in the current study from the EWAS Catalog (http://ewascatalog.org/; accessed on December 1, 2023). Similarly, we annotated the identified CpGs to the nearest genes using Illumina's Manifest File and searched for the previously reported traits that were associated with the genes harboring these CpGs from the GWAS Catalog (https://www.ebi.ac.uk/gwas/; accessed on December 1, 2023).

To examine genetic influences on the identified CpGs, we report here the broad or narrow sense heritability, as appropriate, calculated using twin data from SATSA and LSADT baseline in a previous publication (Reynolds et al., 2020).

#### 2.4.6 Longitudinal analysis

For the significant CpGs identified in the EWAS meta‐analysis, we assessed their longitudinal associations with the FI using 961 repeated measurements of FI and DNA methylation in the SATSA 450K sample. Linear mixed‐effects models with random intercepts at individual and twin‐pair levels were used for the longitudinal analysis. The models were adjusted for age, sex, smoking, and BMI. We compared the directions of the associations between the cross‐sectional and longitudinal associations in SATSA 450K to examine the robustness and temporal stability of the associations.

#### 2.4.7 Comparison with previous EWASs of frailty

Finally, we searched the literature on the previously reported frailty‐associated CpGs and examined whether these sites were also associated with the FI in SATSA and LSADT. We highlighted CpGs that were directionally consistent and with *p* < 0.05 in the EWAS meta‐analysis.

All the analyses were performed using R v.4.2.3.

## 3 RESULTS

### 3.1 Sample characteristics

Baseline characteristics of the four samples used in the EWAS meta‐analysis are displayed in Table 1 and Table S2. The mean age of the SATSA 450K, SATSA EPIC, LSADT 1997, and LSADT 2007 samples was 69.1, 66.4, 78.5, and 86.1 years, and the proportions of women were 60.7%, 54.8%, 69.4%, and 72.1%, respectively (Table 1). The FIs in SATSA 450K, SATSA EPIC, and LSADT 1997 were similar in distribution (mean around 0.1), while the LSADT 2007, which is a follow‐up sample had, on average, a higher FI score (mean 0.161; Table 1 and Figure S1). Frailty generally increased with age (all *p* < 0.05 except in LSADT 2007) and was higher in women than in men (*p* < 0.05 in SATSA 450K and LSADT 2007). However, frailty did not appear to be associated with smoking or BMI in any of the four samples (all *p* > 0.05; Table S2).

**TABLE 1:** Characteristics of the four samples included in the EWAS meta‐analysis.

Variable	SATSA 450K	SATSA EPIC	LSADT 1997	LSADT 2007
No. of individuals	379	146	304	86
Age, year, mean ± SD	69.1 ± 9.6	66.4 ± 8.6	78.5 ± 3.8	86.1 ± 1.8
Women, *n* (%)	230 (60.7)	80 (54.8)	211 (69.4)	62 (72.1)
Smoking status, *n* (%)				
Never	301 (79.4)	112 (76.7)	106 (34.9)	35 (40.7)
Previous	13 (3.4)	5 (3.4)	77 (25.3)	10 (11.6)
Current	65 (17.2)	29 (19.9)	121 (39.8)	41 (47.7)
BMI, mean ± SD	26.0 ± 4.2	26.3 ± 3.3	24.3 ± 3.8	23.3 ± 3.6
Zygosity, *n* (%)				
MZ	169 (44.6)	21 (14.4)	233 (76.6)	36 (41.9)
DZ	209 (55.1)	125 (85.6)	71 (23.4)	50 (58.1)
Unknown	1 (0.3)	–	–	–
FI, mean ± SD	0.101 ± 0.087	0.094 ± 0.075	0.099 ± 0.082	0.161 ± 0.094
FI, median (IQR)	0.083 (0.036–0.137)	0.083 (0.048–0.113)	0.081 (0.035–0.140)	0.146 (0.090–0.222)
FI categories, *n* (%)				
Non‐frail (≤0.1)	221 (58.3)	92 (63.0)	188 (61.8)	30 (34.9)
Pre‐frail (>0.1–0.21)	117 (30.9)	43 (29.5)	87 (28.6)	33 (38.4)
Frail (>0.21)	41 (10.8)	11 (7.5)	29 (9.5)	23 (26.7)

*Note*: The SATSA 450K and SATSA EPIC samples were independent samples of which DNA methylation data were measured using the Illumina's Infinium HumanMethylation450K and MethylationEPIC array, respectively. The LSADT 1997 sample refers to the baseline data and LSADT 2007 is a follow‐up cohort of LSADT 1997; all DNA methylation data in LSADT were measured using the Infinium HumanMethylation450K array. Sample characteristics by frailty groups are provided in Table S2.

Abbreviations: BMI, body mass index; DZ, dizygotic; FI, frailty index; IQR, interquartile range; LSADT, Longitudinal Study of Aging Danish Twins; MZ, monozygotic; SATSA, Swedish Adoption/Twin Study of Aging; SD, standard deviation.

### 3.2 Association between frailty and DNA methylation

**FIGURE 1:**
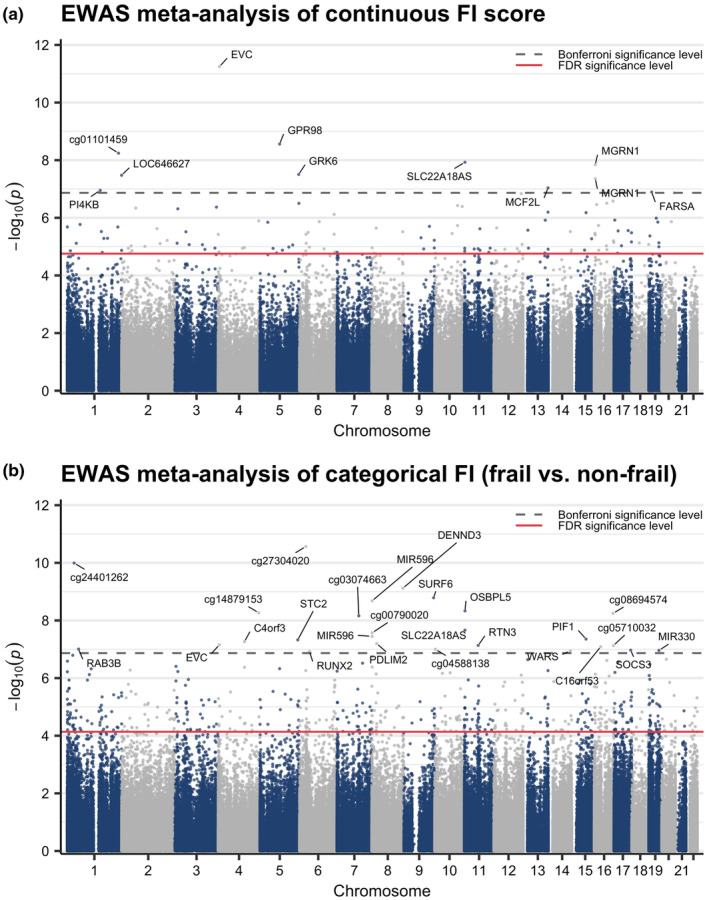
Manhattan plots for the EWAS meta‐analysis of (a) the continuous FI score (per 10% increase) and (b) the categorical FI (frail vs. non‐frail). Estimates were obtained from the fixed‐effect inverse variance weighted meta‐analysis of the SATSA 450K, SATSA EPIC, LSADT 1997, and LSADT 2007 EWAS results. The y‐axis shows the negative log of the associated *p*‐value. A total of 129 and 538 CpGs were considered as statistically significantly associated with the continuous and categorical FI, respectively, with an FDR <0.05 (as shown by all the dots above the solid, red horizontal line). The labeled dots above the dashed, grey horizontal line represent the Bonferroni‐significant CpGs at *p* < 1.36E‐7 (i.e., 0.05/368,249). *EWAS*, epigenome‐wide association study; *FI*, frailty index; *LSADT*, Longitudinal Study of Aging Danish Twins; SATSA, Swedish Adoption/Twin Study of Aging.

Of the 368,249 CpGs included in the EWAS meta‐analysis, we identified 129 CpGs statistically significantly (FDR <0.05) associated with the continuous FI score (Figure 1a); considerable heterogeneity and inconsistency (*I* ^2^ > 75%) were observed for 10 (7.8%) of these sites (Table 2 and Table S3). When additionally considering the FI as a categorical variable with non‐frail as the reference category, no CpG was significantly associated with pre‐frailty, while 538 CpGs were associated with frailty at FDR <0.05 and 34 of which showed considerable heterogeneity (*I* ^2^ > 75%; results for frail vs. non‐frail are shown in Figure 1b, Table 2, and Table S4). Together, there were 78 CpGs significantly associated with both the continuous FI and categorical FI (frail vs. non‐frail). The top CpGs with the smallest *p*‐value associated with the continuous and categorical FI were cg05582310 (mapped to *EVC*) and cg27304020 (N/A), respectively, both of which were hypomethylated with higher frailty scores (Table 2 and Tables S3 and S4).

**TABLE 2:** Top 10 CpGs associated with the continuous FI score and the categorical FI (frail vs. non‐frail) from the EWAS meta‐analysis.

CpG	Gene	Chr	Position^a^	Gene region	Relation to CpG Island	*β*‐coefficient^b^	SE	*p*	FDR	Direction^c^	*I* ^2^ (%)
Association of DNA methylation with continuous FI score											
cg05582310	*EVC*	4	5,760,808	Body	Open sea	−0.007	0.001	5.7 × 10^−12^	2.1 × 10^−6^	−−−−	77.3
cg03607415	*GPR98*	5	89,881,642	Body	Open sea	−0.006	0.001	2.8 × 10^−9^	5.1 × 10^−4^	−−−−	4.9
cg01101459	*−*	1	234,871,477	IGR	Open sea	0.010	0.002	5.7 × 10^−9^	7.0 × 10^−4^	++++	0
cg18458509	*SLC22A18AS*	11	2,920,189	Body	North shelf	−0.006	0.001	1.2 × 10^−8^	0.001	−−−−	0
cg07248377	*MGRN1*	16	4,732,406	Body	Island	0.010	0.002	1.4 × 10^−8^	0.001	++++	53.1
cg19459094	*GRK6*	5	176,856,845	Body	South shelf	−0.005	0.001	3.1 × 10^−8^	0.002	−−−−	21.5
cg16824319	*LOC646627*	1	248,903,933	TSS1500	Open sea	0.006	0.001	3.3 × 10^−8^	0.002	++++	0
cg03963853	*MGRN1*	16	4,732,369	Body	Island	0.007	0.001	4.4 × 10^−8^	0.002	++++	0
cg22004422	*MCF2L*	13	113,740,730	Body	North shelf	−0.004	0.001	9.2 × 10^−8^	0.004	−+−−	64.6
cg06122613	*PI4KB*	1	151,297,900	Body	North shelf	0.010	0.002	1.1 × 10^−7^	0.004	++++	0
Association of DNA methylation with categorical FI (frail vs. non‐frail)											
cg27304020	*−*	6	28,956,327	IGR	Island	−0.029	0.004	2.7 × 10^−11^	1.0 × 10^−5^	−−−−	74.1
cg24401262	*−*	1	31,956,405	IGR	Open sea	−0.016	0.003	1.0 × 10^−10^	1.9 × 10^−5^	−−−+	88.8
cg24476153	*DENND3*	8	142,139,938	5'UTR	South shore	−0.008	0.001	7.6 × 10^−10^	9.3 × 10^−5^	−−−−	27.7
cg14047548	*SURF6*	9	136,199,066	Body	Island	0.002	0.000	1.6 × 10^−9^	1.5 × 10^−4^	+++−	0
cg25673948	*MIR596*	8	1,765,296	TSS200	South shore	−0.020	0.003	2.1 × 10^−9^	1.5 × 10^−4^	−−−−	73.5
cg07992377	*OSBPL5*	11	3,173,953	5'UTR	Open sea	0.012	0.002	4.6 × 10^−9^	2.6 × 10^−4^	+−++	45.4
cg14879153	*−*	4	185,459,316	IGR	Open sea	−0.005	0.001	5.4 × 10^−9^	2.6 × 10^−4^	+−−+	14.7
cg08694574	*−*	16	86,011,615	IGR	South shelf	0.015	0.003	5.7 × 10^−9^	2.6 × 10^−4^	++++	21.2
cg03074663	*−*	7	99,728,719	IGR	South shelf	−0.003	0.000	6.9 × 10^−9^	2.8 × 10^−4^	−−−+	2.5
cg18458509	*SLC22A18AS*	11	2,920,189	Body	North shelf	−0.015	0.003	2.2 × 10^−8^	8.0 × 10^−4^	+−−−	39.4

*Note*: Listed are the top CpGs ranked by *p*‐values that were associated with the FI at FDR <0.05 from the EWAS meta‐analysis. Estimates for all the significant CpGs (FDR <0.05) are provided in Tables S3 and S4.

Abbreviations: Chr, chromosome; FDR, false discovery rate; FI, frailty index; *I* ^2^; heterogeneity; IGR, intergenic region; LSADT, Longitudinal Study of Aging Danish Twins; SATSA, Swedish Adoption/Twin Study of Aging; SE, standard error; TSS, transcription start site; UTR, untranslated region.

^a^ Based on genome assembly GRCh37 (hg19).

^b^ The *β*‐coefficients represent changes in DNA methylation level for a 10% increase in the FI (for models using the continuous FI score as the independent variable) or comparing frail vs. non‐frail groups (for models using the categorical FI as the independent variable). The EWAS models in each cohort were controlled for age, sex, smoking, BMI, cellular compositions, batch effect, and accounted for twin relatedness by cluster robust standard errors. Estimates for the four samples were combined using fixed‐effect inverse variance weighted meta‐analysis.

^c^ Order of the included samples: SATSA 450K, SATSA EPIC, LSADT 1997, and LSADT 2007. “+” represents a positive association and “−“represents an inverse association between DNA methylation and the FI.

The top GO term identified in the pathway analysis was regulatory RNA binding, and the top KEGG term was the Notch signaling pathway, although none of the GO terms or KEGG pathways were enriched at FDR <0.05 (Table S5).

Furthermore, we identified a DMR in the *MGRN1* gene that was positively associated with the continuous FI and two DMRs in *MIR596* and *TAPBP* that were negatively associated with the categorical FI after Bonferroni adjustment (Table 3).

**TABLE 3:** Top 10 DMRs associated with the continuous FI score and the categorical FI (frail vs. non‐frail) from the EWAS meta‐analysis.

Chr	Start position^a^	End position^a^	Gene	Number of CpGs	*β*‐Coefficient	SE	*p*	Bonferroni‐adjusted *p*
Top 10 DMRs associated with continuous FI								
**16**	**4,732,369**	**4,732,406**	***MGRN1***	**2**	**0.168**	**0.032**	**1.8 × 10** ^**−7**^	**0.028**
16	4,731,639	4,732,262	*MGRN1*	4	0.108	0.026	2.4 × 10^−5^	1
15	79,298,209	79,298,691	*RASGRF1*	8	0.077	0.019	3.8 × 10^−5^	1
20	42,142,559	42,142,673	*L3MBTL*	4	−0.120	0.031	1.1 × 10^−4^	1
7	94,285,270	94,285,814	*SGCE*	12	−0.059	0.016	2.1 × 10^−4^	1
6	30,296,700	30,297,627	*TRIM39*	6	0.057	0.016	2.3 × 10^−4^	1
8	1,765,312	1,765,358	*MIR596*	2	−0.133	0.036	2.4 × 10^−4^	1
20	42,142,751	42,142,784	*L3MBTL*	3	−0.111	0.031	3.0 × 10^−4^	1
6	31,869,534	31,869,572	*ZBTB12*	5	−0.083	0.024	4.7 × 10^−4^	1
6	31,762,555	31,762,644	*VARS*	3	−0.107	0.034	1.6 × 10^−3^	1
Top 10 DMRs associated with categorical FI (frail vs. non‐frail)								
**8**	**1,765,217**	**1,765,820**	***MIR596***	**7**	**−0.421**	**0.067**	**3.6 × 10** ^**−10**^	**5.5 × 10** ^**−5**^
**6**	**33,282,313**	**33,283,162**	***TAPBP***	**12**	**−0.297**	**0.053**	**2.3 × 10** ^**−8**^	**0.004**
7	27,142,437	27,143,046	*HOXA2*	5	−0.364	0.072	3.8 × 10^−7^	0.058
15	79,298,209	79,298,678	*RASGRF1*	7	0.199	0.048	2.8 × 10^−5^	1
22	45,608,440	45,608,516	*C22orf9*	5	0.293	0.072	5.4 × 10^−5^	1
20	42,142,751	42,142,784	*L3MBTL*	3	−0.350	0.091	1.2 × 10^−4^	1
20	42,142,559	42,142,673	*L3MBTL*	4	−0.313	0.090	4.9 × 10^−4^	1
17	33,759,512	33,759,965	*SLFN12*	6	−0.333	0.097	6.0 × 10^−4^	1
7	27,143,334	27,143,403	*HOXA2*	2	−0.359	0.106	7.0 × 10^−4^	1
6	32,121,393	32,121,420	*PPT2*	2	−0.265	0.087	2.5 × 10^−3^	1

*Note*: DMRs were identified using the *dmrff* R package. Bolded is the 3 DMRs with an adjusted *p* < 0.05 using the Bonferroni correction.

Abbreviations: Chr, chromosome; DMR, differentially methylated region; FI, frailty index; SATSA, Swedish Adoption/Twin Study of Aging; SE, standard error.

^a^ Based on genome assembly GRCh37 (hg19).

### 3.3 Look‐ups of the findings

We queried the EWAS Catalog and found many of our 589 identified CpG sites were previously shown to be associated with chronological age (455 sites) and age‐related diseases such as chronic obstructive pulmonary disease (85 sites), rheumatoid arthritis (72 sites), type 2 diabetes (71 sites), and clear cell renal carcinoma (69 sites) (Table S6). As many frailty‐associated CpGs may also be associated with chronological age, we examined the association between age and the significant sites in our EWAS models in the SATSA 450K sample. After adjusting for frailty, sex, smoking, and BMI, 55 of the 589 sites (9.3%) were associated with chronological age at *p* < 0.05 (Table S7). We also found in the GWAS Catalog that many of the genes that our frailty‐associated CpGs mapped to have been previously linked to body height, educational attainment, and BMI (Table S6).

To examine genetic influences on these CpGs, we looked up the twin‐based heritability of the 589 identified CpGs from a previous analysis in the SATSA and LSADT cohorts (Reynolds et al., 2020). Of the 577 available CpGs, the mean heritability at baseline was 17.7% (range 0%–86.8%; Table S8).

### 3.4 Longitudinal analysis

For the 589 CpGs that were associated with either the continuous or the categorical FI in the EWAS meta‐analysis, we analyzed their longitudinal associations with the FI in the SATSA 450K sample, in which the individuals participated on average in two IPT waves (interquartile range 1–4) over a maximum of 20 years (Table S9). Overall, we observed mostly consistent directions of the associations when comparing the cross‐sectional vs. longitudinal estimates in the SATSA 450K sample (*r* = 0.79 for continuous FI and *r* = 0.76 for frail vs. non‐frail; Figure S2).

### 3.5 Comparison with previous EWASs of frailty

Lastly, we performed a literature search and identified 80 CpGs previously reported to be associated with frailty in different cohorts (Gale et al., 2018; Gao et al., 2017; Kim et al., 2018; Li et al., 2022). Of the 77 available CpGs, 34 sites (44.2%) were also associated with the FI in our EWAS meta‐analysis at *p* < 0.05 and had the same direction of association as previously reported (Table S10).

## 4 DISCUSSION

In this study, we performed a meta‐analysis of EWASs of frailty in four samples of Swedish and Danish twins and identified 589 CpGs and three DMRs statistically significantly associated with either the continuous or categorical FI. Many of these CpGs were also associated with the FI longitudinally, and we replicated 34 of the 77 previously reported frailty‐associated CpGs (at *p* < 0.05).

Adding to the literature, we report 589 novel CpGs at FDR <0.05 in the meta‐analysis after adjusting for age, sex, and other confounders; none of these sites have previously been associated with frailty. As DNA methylation can change throughout the lifespan and is responsive to environmental factors, we additionally examined the longitudinal associations between the CpGs identified in the cross‐sectional analysis in SATSA. The consistency between the cross‐sectional and longitudinal findings indicates that the associations are robust and relatively stable over age. Moreover, we identified three DMRs in the *MGRN1* (Mahogunin Ring Finger‐1), *MIR596* (microRNA‐596), and *TAPBP* (tapasin) genes, where *MGRN1* was hypomethylated and *MIR596* and *TAPBP* were hypermethylated with increased FI. MGRN1 is an E3 ubiquitin ligase and its decreased levels have been linked to neurodegeneration and neuronal aging (Benvegnù et al., 2017; Chhangani & Mishra, 2013). A previous study showed that hypomethylation in the *MGRN1* gene was associated with breast cancer (Tang et al., 2016). MicroRNA‐596 plays an important role in modulation of tumor growth, differentiation, and proliferation (Fu et al., 2020; Liu et al., 2018; Zhang & Dai, 2019). DNA hypermethylation in the *MIR596* gene has been linked to several cancers, such as oral cancer (Endo et al., 2013), acute myeloid leukemia (Huang et al., 2018), and hepatocellular carcinoma (Anwar et al., 2013). Tapasin is a glycoprotein that assists in the assembly and loading of peptides onto major histocompatibility complex class I molecules, playing a crucial role in the immune system's antigen presentation pathway (Ortmann et al., 1997). Hypermethylation in the *TAPBP* gene has been associated with obesity (Martin et al., 2019) and insulin sensitivity in early childhood (van Dijk et al., 2018). Altogether, these findings suggest that DNA methylation in genes that are related to neuronal pathways, cancer development, and immune signaling is associated with frailty. These results are also in line with a previous EWAS of the FI that has identified several CpGs located in genes that have links to cancers and neurodegenerative disorders (Li et al., 2022). Our results also concur with findings from a large GWAS demonstrating that genetic variants in neurological/brain function pathways underlie the risk of frailty (Atkins et al., 2021).

To date, there have only been a limited number of EWASs on frailty, and none of the reported CpGs have been consistently associated with frailty across different cohorts (Gale et al., 2018; Kim et al., 2018; Li et al., 2022; Livshits, Malkin, et al., 2018). Compared to genome‐wide association studies, EWASs on age‐related diseases generally have a lower level of replicability as they typically have smaller sample sizes and can be more easily affected by the differences in covariate adjustment strategies, significance thresholds, and study designs in different studies (Hillary et al., 2023). In particular, as frailty is a multifactorial syndrome reflecting the decline in multiple physiological and organ systems during aging (Mak et al., 2023) and the epigenome is also closely linked to age (Wang et al., 2018) and age‐related diseases (Hillary et al., 2023), it is possible that many CpGs that associate with (biological) aging and diseases can also influence frailty. Thus, it may be difficult to identify CpGs that are specific to frailty. This was also indicated by our look‐up analysis in the EWAS Catalog, in which most of our identified CpGs were previously reported to be associated with chronological age and age‐related traits, such as chronic obstructive pulmonary disease, rheumatoid arthritis, and type 2 diabetes. Nevertheless, we found that age was not significantly associated with most of our identified CpGs after accounting for frailty in the EWAS models, suggesting that these CpGs may be more strongly related to frailty than chronological age. We were also able to replicate 44.2% of the frailty‐associated CpGs reported in the literature, further strengthening the validity of these findings.

The major strengths of this study include the use of two well‐established Swedish and Danish twin cohorts with validated measures of frailty and DNA methylation. The longitudinal data also allowed us to assess the temporal stability of the associations. However, several limitations should be acknowledged. It should be noted that our participants were of European ancestry, and thus our results may not be generalizable to other populations or ethnic groups. Due to the relatively small sample size, we lacked the statistical power to perform further stratified analysis for example by sex. As our EWAS analysis was limited to CpGs that passed quality control in SATSA and LSADT, potential associations with CpGs that were not included in our analysis may have been missed. Moreover, the non‐identical deficit items included in FIs in SATSA and LSADT may have affected the results and contributed to the heterogeneity (*I* ^2^) between the studies. Finally, as we only included the FI as a measure of frailty, it would be important for future studies to examine whether other measures of frailty, such as the physical frailty phenotype, may also be associated with our identified CpGs.

In conclusion, in this study, we identified 589 novel CpG sites and 3 DMRs that were statistically significantly associated with the FI in two European twin cohorts with the results strengthening especially the role of brain pathway genes in frailty. The results warrant further investigation in other populations to test whether DNA methylation at these sites could serve as potential biomarkers of frailty.

## AUTHOR CONTRIBUTIONS

JKLM, ACS, SH, MS, LK, and JJ contributed to the study design and statistical analysis plan. JKLM, ACS, MN, YW, IKK, and MS contributed to data curation. JKLM and ACS performed the analyses. NLP is the founder and principal investigator of the SATSA study. CAR, RK‐H, SH, and JJ were involved in supervision. JKLM and JJ drafted the manuscript. All authors contributed to interpretation of the results, read, and approved the final manuscript.

## FUNDING INFORMATION

The Swedish Adoption/Twin Study of Aging was supported by the National Institutes of Health (NIH; Grants AG04563 and AG10175), the MacArthur Foundation Research Network on Successful Aging, the Swedish Research Council for Working Life and Social Research (FAS; Grants 97:0147:1B, 2009‐0795), and the Swedish Research Council (825‐2007‐7460 and 825‐2009‐6141). The Longitudinal Study of Aging Danish Twins and the data used were supported by the National Institutes of Health, National Institute on Aging grant P01 AG08761, and the European Union's Seventh Framework Programme (FP7/2007–2011), while the present work was also supported by the Fabrikant Vilhelm Pedersen og Hustrus Legat on recommendation by the Novo Nordisk Foundation. This work was also supported by the Swedish Research Council (2018‐02077, 2019‐01272, 2020‐06101), the Loo and Hans Osterman Foundation for Medical Research, FORTE (the Swedish Research Council for Health, Working Life, and Welfare), the Sigrid Jusélius Foundation, the Strategic Research Program in Epidemiology at Karolinska Institutet, the Karolinska Institutet Foundations, the King Gustaf V and Queen Victoria's Foundation of Freemasons, the Yrjö Jahnsson Foundation (grant no 20217416), the Juho Vainio Foundation (grant number 202100335), the Päivikki and Sakari Sohlberg Foundation (grant number 220032), and the Tampere Tuberculosis Foundation.

## CONFLICT OF INTEREST STATEMENT

The authors declare no conflict of interest.

## Supporting information


Appendix S1.

## Data Availability

For the SATSA study, methylation data are available in the EMBL‐EBI under accession number S‐BSST1206 (https://www.ebi.ac.uk/biostudies/studies/S‐BSST1206), and phenotypic data are available in the National Archive of Computerized Data on Aging under accession number ICPSR 3843 (https://www.icpsr.umich.edu/web/NACDA/studies/3843). For the LSADT study, according to Danish legislation, transfer and sharing of individual‐level data requires prior approval from the Danish Data Protection Agency and requires that data sharing requests be dealt with on a case‐by‐case basis. Therefore, the data cannot be deposited in a public database. However, we welcome any inquiries regarding collaboration and individual requests for data sharing. EWAS summary statistics are available at https://doi.org/10.6084/m9.figshare.25239475.v1.
