# Supplementary material for: Epigenome‐wide analysis of frailty: Results from two European twin cohorts
Source: Aging Cell. 2024 Feb 27;23(6):e14135. doi: 10.1111/acel.14135 (PMC11166364; doi:10.1111/acel.14135)
Supplement: Supplementary file 1 — Appendix S1. [file ACEL-23-e14135-s001.zip › ACE-23-0795_supplement_1.docx]

**Supplement 1: Supplementary Figures 1 & 2, Supplementary Tables 1, 2, 5, 9**

[Supplementary Figure 1. Distribution of the frailty index in the four samples included in the EWAS meta-analysis. 2](#_Toc153187529)

[Supplementary Figure 2. Comparison of cross-sectional (x-axis) and longitudinal (y-axis) estimates for the associations between DNA methylation level of the identified CpGs and the (a) continuous FI score (per 10% increase) and (b) categorical FI (frail vs. non-frail) in the SATSA 450K sample. 3](#_Toc153187530)

[Supplementary Table 1. List of deficit items and coding of the frailty index in the SATSA and LSADT samples 4](#_Toc153187531)

[Supplementary Table 2. Participant characteristics of the four samples included in EWAS meta-analysis stratified by FI categories. 7](#_Toc153187532)

[Supplementary Table 5. Top 20 GO terms and KEGG pathways identified from the pathway analysis 8](#_Toc153187533)

[Supplementary Table 10. Associations between the previously reported frailty-associated CpGs and the FI in the EWAS meta-analysis 9](#_Toc153187534)

| **a** | 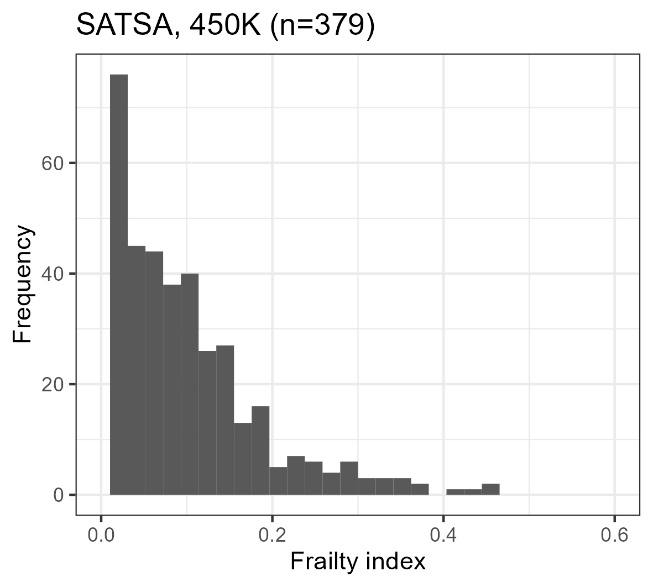 | **b** | 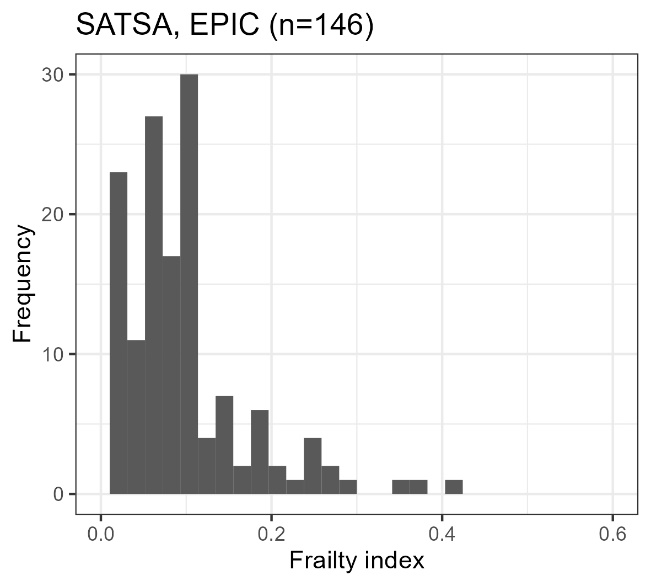 |
| --- | --- | --- | --- |
| **c** | 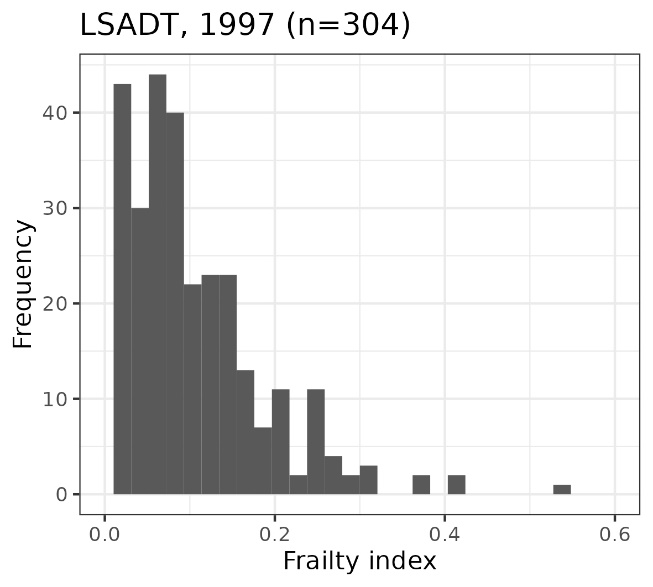 | **d** | 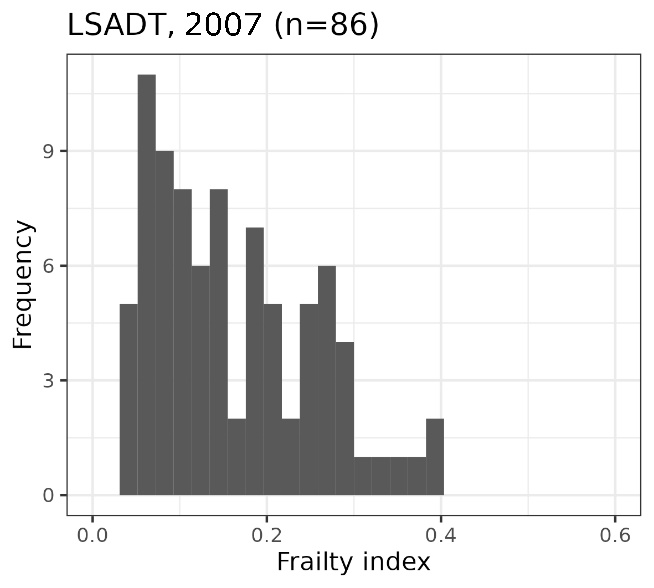 |

# Supplementary Figure 1. Distribution of the frailty index in the four samples included in the EWAS meta-analysis.

*EWAS*, epigenome-wide association study; *FI*, frailty index; *LSADT*, Longitudinal Study of Aging Danish Twins; *SATSA*, Swedish Adoption/Twin Study of Aging. The SATSA 450K and SATSA EPIC samples were independent samples of which DNA methylation data were measured using the Illumina’s Infinium HumanMethylation450K and MethylationEPIC array, respectively. The LSADT 1997 sample refers to the baseline data and LSADT 2007 is a follow-up cohort of LSADT 1997; all DNA methylation data in LSADT were measured using the Infinium HumanMethylation450K array.

**
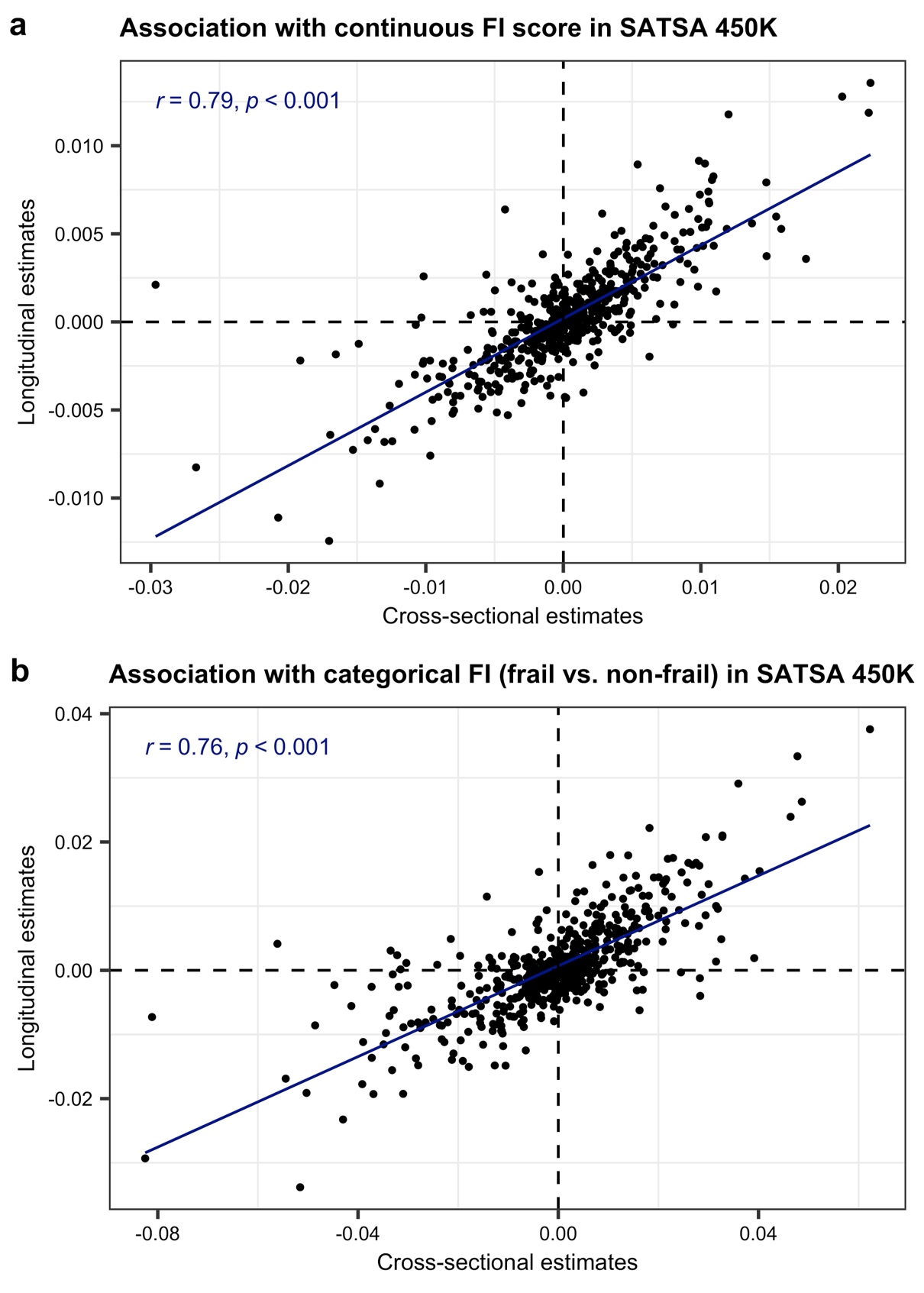
**

# Supplementary Figure 2. Comparison of cross-sectional (x-axis) and longitudinal (y-axis) estimates for the associations between DNA methylation level of the identified CpGs and the (a) continuous FI score (per 10% increase) and (b) categorical FI (frail vs. non-frail) in the SATSA 450K sample.

The 589 dots in each panel represent the CpGs identified from the meta-analysis (i.e., associated with either the continuous or categorical FI at FDR <.05). The blue regression line and the Pearson’s correlation coefficient suggest a strong positive correlation between the estimates. Cross-sectional estimates (first available measurement per person in the SATSA 450K sample) were obtained from generalized estimating equation models adjusted for age, sex, smoking, and BMI, and accounted for twin-relatedness using cluster robust standard errors. Longitudinal estimates (all available measurements in the SATSA 450K sample) were obtained from linear mixed models adjusted for age, sex, smoking, BMI, and with random intercepts at individual and twin pair levels. *EWAS*, epigenome-wide association study; *FI*, frailty index; *SATSA*, Swedish Adoption/Twin Study of Aging.

# Supplementary Table 1. List of deficit items and coding of the frailty index in the SATSA and LSADT samples.

| Items | SATSA 450K and EPIC (42 items) | LSADT 1997 (43 items) | LSADT 2007 (36 items) |
| --- | --- | --- | --- |
| Self-reported general health | Good=0, Mediocre=0.5, Bad=1 | Very good=0, Good=0.25, Fair=0.5, Poor=0.75, Very poor=1 | Very good=0, Good=0.25, Fair=0.5, Poor=0.75, Very poor=1 |
| Cataracts | No=0, Yes=1 | No=0, Yes=1 | No=0, Yes=1 |
| Glaucoma | - | No=0, Yes=1 | No=0, Yes=1 |
| Sclerosis in eye | - | - | No=0, Yes=1 |
| Thrombosis in eye | - | - | No=0, Yes=1 |
| Migraine | - | No=0, Yes=1 | No=0, Yes=1 |
| Vision status | Perfect=0, Good=0.25, Pretty Good=0.5, Bad=0.75, Blind or almost blind=1 | - | - |
| Hearing status | Perfect=0, Good=0.25, Pretty Good=0.5, Bad=0.75, Deaf or almost deaf=1 | - | - |
| Cancer or leukemia | No=0, Yes=1 | No=0, Yes=1 | No=0, Yes=1 |
| Rheumatoid arthritis | No=0, Yes=1 | No=0, Yes=1 | No=0, Yes=1 |
| Gout | No=0, Yes=1 | No=0, Yes=1 | No=0, Yes=1 |
| Chronic bronchitis or emphysema | No=0, Yes=1 | No=0, Yes=1 | No=0, Yes=1 |
| Chest pain | No=0, Yes=1 | - | - |
| Circulation problems in arms or legs | No=0, Yes=1 | No=0, Yes=1 | No=0, Yes=1 |
| Fluid in lungs | - | - | No=0, Yes=1 |
| Persistent cough | No=0, Yes=1 | - | - |
| Diabetes | No=0, Yes=1 | No=0, Yes=1 | No=0, Yes=1 |
| Goiter or other gland problems | No=0, Yes=1 | - | - |
| Gallstone |  |  | No=0, Yes=1 |
| Angina pectoris | - | No=0, Yes=1 | No=0, Yes=1 |
| Irregular heart rhythm | - | No=0, Yes=1 | No=0, Yes=1 |
| Heart failure/other heart problems | No=0, Yes=1 | No=0, Yes=1 | No=0, Yes=1 |
| Heart attack | - | No=0, Yes=1 | No=0, Yes=1 |
| Hypertension | No=0, Yes=1 | No=0, Yes=1 | No=0, Yes=1 |
| Kidney disease | No=0, Yes=1 | No=0, Yes=1 | No=0, Yes=1 |
| Kidney stones | - | - | No=0, Yes=1 |
| Brittle bones | No=0, Yes=1 | - | - |
| Fractured femur or hip | - | No=0, Yes=1 | - |
| Fractured spine | - | No=0, Yes=1 | - |
| Osteoarthritis | - | No=0, Yes=1 | No=0, Yes=1 |
| Osteoporosis | - | No=0, Yes=1 | No=0, Yes=1 |
| Sciatica | No=0, Yes=1 | - | - |
| Anemia | No=0, Yes=1 | - | - |
| Cerebral hemorrhage or blood clot in brain | No=0, Yes=1 | - | - |
| Stroke | - | No=0, Yes=1 | No=0, Yes=1 |
| Have or have had paralysis of arms or legs | - | No=0, Yes=1 | - |
| Parkinson’s disease | - | No=0, Yes=1 | No=0, Yes=1 |
| Epilepsy | - | No=0, Yes=1 | No=0, Yes=1 |

**Supplementary Table 1.** (*continued*)

| Items | SATSA 450K and EPIC (42 items) | LSADT 1997 (43 items) | LSADT 2007 (36 items) |
| --- | --- | --- | --- |
| Dizziness | No=0, Yes=1 | No=0, Yes=1 | - |
| Fainting fits | - | No=0, Yes=1 | - |
| Gastric ulcer | No=0, Yes=1 | - | - |
| Allergies/allergic manifestations | No=0, Yes=1 | - | - |
| Asthma | No=0, Yes=1 | No=0, Yes=1 | No=0, Yes=1 |
| Health prevents from doing things normally would like to do | No=0, Somewhat=0.5, Yes=1 | - |  |
| Feel well enough to do what want | - | Yes, always=0, Yes, almost always=0.25, Yes, sometimes=0.5, No, hardly ever=0.75, No, never=1 | - |
| Getting home care | - | Never=0, Less than weekly=0.25, Weekly=0.5, 2-3 times per week=0.75, Every day=1 | - |
| Able to read normal newspaper text | - | Yes, without difficulty=0, Yes, with minor difficulty=0.25, Yes, with major difficulty=0.75, No=1 | Yes, without difficulty=0, Yes, with minor difficulty=0.25, Yes, with major difficulty=0.75, No=1 |
| Able to hear what is said in a normal conversation with 3 or more persons | - | Yes, without difficulty=0, Yes, with minor difficulty=0.25, Yes, with major difficulty = 0.75, No=1 | - |
| Shower and bathe | No problem=0, Needs help=0.5, Cannot=1 | - | - |
| Get in and out of bed | No problem=0, Needs help=0.5, Cannot=1 | - | - |
| Able to get up from a chair and a bed | - | Yes=0, Needs help=0.5, No=1 | Yes=0, Needs help=0.5, No=1 |
| Able to climb one flight of stairs without resting | - | Yes, without difficulty=0, Yes, with minor difficulty=0.25, Yes, with major difficulty=0.75, No=1 | Yes, without difficulty=0, Yes, with minor difficulty=0.25, Yes, with major difficulty=0.75, No=1 |
| Dress and undress | No problem=0, Needs help=0.5, Cannot=1 | - | - |
| Self-grooming | No problem=0, Needs help=0.5, Cannot=1 | - | - |
| Walking | No problem=0, Needs help=0.5, Cannot=1 | - | - |
| Able to walk 400 meters without resting | - | Yes, without difficulty=0, Yes, with minor difficulty=0.25, Yes, with major difficulty=0.75, No=1 | Yes, without difficulty=0, Yes, with minor difficulty=0.25, Yes, with major difficulty=0.75, No=1 |
| Able to go to the toilet | No problem=0, Trouble getting to toilet in time=1 | Yes=0, Needs help=0.5, No=1 | Yes=0, Needs help=0.5, No=1 |
| Able to carry a 5 kg object | - | Yes, without difficulty=0, Yes, with minor difficulty=0.25, Yes, with major difficulty=0.75, No=1 | Yes, without difficulty=0, Yes, with minor difficulty=0.25, Yes, with major difficulty=0.75, No=1 |

**Supplementary Table 1.** (*continued*)

| Items | SATSA 450K and EPIC (42 items) | LSADT 1997 (43 items) | LSADT 2007 (36 items) |
| --- | --- | --- | --- |
| Travel further distances | Can travel alone=0, Can go by taxi=0.5, Needs helper, special assistance or doesn’t travel=1 | - | - |
| Housework | No problems=0, Needs help=0.5, Doesn’t do=1 | - | - |
| Prepare meals | Can plan/prepare=0, Can heat up=0.5, Doesn’t cook=1 | - | - |
| Manage medications | No problems=0^,^ Needs help=0.5, Doesn’t do=1 | - | - |
| Manage money | No problems=0, Needs help=0.5, Doesn’t do=1 | - | - |
| Use telephone | Can look up numbers and dial=0, Needs help or doesn’t use phone=1 | - | - |
| Grocery shopping | Can shop=0, Needs help=0.5, Doesn’t shop=1 | - | - |
| Feeling lonely | Never, almost never, rather seldom=0, Quite often, always, almost always=1 | No=0; Sometimes=0.5; Most of the time=1 | - |
| Feeling depressed | Never, almost never or rather seldom=0, Quite often, always, almost always=1 | - | - |
| Ever been diagnosed with depression | - | No=0, Yes=1 | - |
| Consider oneself happy and carefree | Yes=0, No=1 | Most of the time=0; Sometimes=0.5; Never or hardly ever=1 | - |
| Usually feels tired/ have less energy and find it harder to get things done | No=0, Yes=1 | No=0; Sometimes=0.5; Most of the time=1 | - |
| Any signs of the respondent being confused during the interview (interviewer assessment) | - | No=0, Yes=1 | No=0, Yes=1 |
| Was the interview difficult to perform (interviewer assessment)? | - | - | No=0, Somewhat=0.5, Yes=1 |
| Interview difficult because of cognitive deficits/dementia (interviewer assessment) | - | No=0, Yes=1 | - |
| Interview difficult because of speech impairment (interviewer assessment) | - | No=0, Yes=1 | - |
| Body mass index | - | - | 18.5 to <25=0, 25 to <30=0.5, <18.5 or ≥30=1 |

*LSADT*, Longitudinal Study of Aging Danish Twins; *SATSA*, Swedish Adoption/Twin Study of Aging.

# Supplementary Table 2. Participant characteristics of the four samples included in EWAS meta-analysis stratified by FI categories.

| **Variable** | **SATSA 450K (n=379)** | | | | **SATSA EPIC (n=146)** | | | |
| --- | --- | --- | --- | --- | --- | --- | --- | --- |
|  | **Robust (≤0.1)** | **Prefrail (>0.1-0.21)** | **Frail (>0.21)** | ***p*** | **Robust (≤0.1)** | **Prefrail (>0.1-0.21)** | **Frail (>0.21)** | ***p*** |
| No. of individuals | 221 (58.3) | 117 (30.9) | 41 (10.8) | - | 92 (63.0) | 43 (29.5) | 11 (7.5) | - |
| Age, year, mean ± SD | 67.0 ± 9.3 | 70.2 ± 8.8 | 76.8 ± 8.7 | <0.001 | 65.1 ± 8.3 | 67.3 ± 9.1 | 73.1 ± 5.1 | 0.01 |
| Women, *n* (%) | 114 (51.6) | 83 (70.9) | 33 (80.5) | <0.001 | 50 (54.3) | 22 (51.2) | 8 (72.7) | 0.435 |
| Smoking status, n (%) |  |  |  | 0.378 |  |  |  | 0.18 |
| Never | 171 (77.4) | 100 (85.5) | 30 (73.2) |  | 68 (73.9) | 33 (76.7) | 11 (100.0) |  |
| Previous | 8 (3.6) | 3 (2.6) | 2 (4.9) |  | 5 (5.4) | 0 (0.0) | 0 (0.0) |  |
| Current | 42 (19.0) | 14 (12.0) | 9 (22.0) |  | 19 (20.7) | 10 (23.3) | 0 (0.0) |  |
| BMI, mean ± SD | 25.8 ± 3.5 | 26.4 ± 4.9 | 26.2 ± 4.8 | 0.358 | 26.3 ± 3.1 | 26.1 ± 3.5 | 26.6 ± 4.4 | 0.919 |
| Zygosity, n (%) |  |  |  | 0.469 |  |  |  |  |
| MZ | 104 (47.1) | 49 (41.9) | 16 (39.0) |  | 12 (13.0) | 5 (11.6) | 4 (36.4) | 0.095 |
| DZ | 117 (52.9) | 67 (57.3) | 25 (61.0) |  | 80 (87.0) | 38 (88.4) | 7 (63.6) |  |
| Unknown | 0 (0.0) | 1 (0.9) | 0 (0.0) |  | - | - | - |  |
| FI, mean ± SD | 0.045 ± 0.027 | 0.139 ± 0.029 | 0.294 ± 0.069 | <0.001 | 0.052 ± 0.028 | 0.134 ± 0.034 | 0.290 ± 0.065 | <0.001 |
| FI, median (IQR) | 0.042 (0.024–0.071) | 0.131 (0.113–0.161) | 0.286 (0.238–0.339) | <0.001 | 0.054 (0.030–0.077) | 0.113 (0.107–0.155) | 0.262 (0.247–0.321) | <0.001 |
| **Variable** | **LSADT 1997 (n=304)** | | | | **LSADT 2007 (n=86)** | | | |
|  | **Robust (≤0.1)** | **Prefrail (>0.1-0.21)** | **Frail (>0.21)** | ***p*** | **Robust (≤0.1)** | **Prefrail (>0.1-0.21)** | **Frail (>0.21)** | ***p*** |
| No. of individuals | 188 (61.8) | 87 (28.6) | 29 (9.5) | - | 30 (34.9) | 33 (38.4) | 23 (26.7) | - |
| Age, year, mean ± SD | 78.1 ± 3.5 | 78.5 ± 3.6 | 80.7 ± 5.6 | 0.002 | 86.4 ± 1.9 | 86.1 ± 1.7 | 85.5 ± 1.6 | 0.179 |
| Women, *n* (%) | 128 (68.1) | 61 (70.1) | 22 (75.9) | 0.689 | 17 (56.7) | 25 (75.8) | 20 (87.0) | 0.043 |
| Smoking status, n (%) |  |  |  | 0.32 |  |  |  | 0.83 |
| Never | 66 (35.1) | 28 (32.2) | 12 (41.4) |  | 14 (46.7) | 13 (39.4) | 8 (34.8) |  |
| Previous | 54 (28.7) | 18 (20.7) | 5 (17.2) |  | 4 (13.3) | 4 (12.1) | 2 (8.7) |  |
| Current | 68 (36.2) | 41 (47.1) | 12 (41.4) |  | 12 (40.0) | 16 (48.5) | 13 (56.5) |  |
| BMI, mean ± SD | 24.2 ± 3.6 | 24.7 ± 3.9 | 24.3 ± 5.1 | 0.501 | 22.9 ± 3.9 | 23.7 ± 3.3 | 23.3 ± 3.8 | 0.667 |
| Zygosity, n (%) |  |  |  | 0.412 |  |  |  | 0.803 |
| MZ | 141 (75.0) | 71 (81.6) | 21 (72.4) |  | 14 (46.7) | 13 (39.4) | 9 (39.1) |  |
| DZ | 47 (25.0) | 16 (18.4) | 8 (27.6) |  | 16 (53.3) | 20 (60.6) | 14 (60.9) |  |
| Unknown | - | - | - |  | - | - | - |  |
| FI, mean ± SD | 0.049 ± 0.029 | 0.147 ± 0.030 | 0.284 ± 0.073 | <0.001 | 0.068 ± 0.026 | 0.156 ± 0.035 | 0.288 ± 0.050 | <0.001 |
| FI, median (IQR) | 0.049 (0.023–0.076) | 0.140 (0.122–0.166) | 0.256 (0.238–0.302) | <0.001 | 0.069 (0.057–0.090) | 0.150 (0.132–0.193) | 0.278 (0.250–0.299) | <0.001 |

*BMI*, body mass index; *DZ*, dizygotic; *EWAS*, epigenome-wide association study; *FI*, frailty index; *IQR*, interquartile range; *LSADT*, Longitudinal Study of Aging Danish Twins; *MZ*, monozygotic; *SATSA*, Swedish Adoption/Twin Study of Aging; *SD*, standard deviation. The SATSA 450K and SATSA EPIC samples were independent samples of which DNA methylation data were measured using the Illumina’s Infinium HumanMethylation450K and MethylationEPIC array, respectively. The LSADT 1997 sample refers to the baseline data and LSADT 2007 is a follow-up cohort of LSADT 1997; all DNA methylation data in LSADT were measured using the Infinium HumanMethylation450K array.

# Supplementary Table 5. Top 20 GO terms and KEGG pathways identified from the pathway analysis.

| **Term** | **Description** | **Ontology** | **No. of genes** | **No. of genes differentially methylated** | ***p*** | **FDR** |
| --- | --- | --- | --- | --- | --- | --- |
| **GO term** | | | | | | |
| GO:0061980 | regulatory RNA binding | Molecular function | 45 | 7 | 1E-04 | 1 |
| GO:0031584 | activation of phospholipase D activity | Biological process | 6 | 3 | 4E-04 | 1 |
| GO:0004321 | fatty-acyl-CoA synthase activity | Molecular function | 6 | 2 | 0.001 | 1 |
| GO:0034162 | toll-like receptor 9 signaling pathway | Biological process | 17 | 4 | 0.001 | 1 |
| GO:0070578 | RISC-loading complex | Cellular component | 8 | 3 | 0.001 | 1 |
| GO:0032621 | interleukin-18 production | Biological process | 12 | 3 | 0.001 | 1 |
| GO:0032661 | regulation of interleukin-18 production | Biological process | 12 | 3 | 0.001 | 1 |
| GO:0042730 | fibrinolysis | Biological process | 27 | 4 | 0.001 | 1 |
| GO:0090557 | establishment of endothelial intestinal barrier | Biological process | 9 | 3 | 0.001 | 1 |
| GO:0097546 | ciliary base | Cellular component | 44 | 6 | 0.002 | 1 |
| GO:0035197 | siRNA binding | Molecular function | 8 | 3 | 0.002 | 1 |
| GO:0003951 | NAD+ kinase activity | Molecular function | 2 | 2 | 0.002 | 1 |
| GO:0006741 | NADP biosynthetic process | Biological process | 2 | 2 | 0.002 | 1 |
| GO:0035198 | miRNA binding | Molecular function | 34 | 5 | 0.002 | 1 |
| GO:0051851 | modulation by host of symbiont process | Biological process | 75 | 7 | 0.002 | 1 |
| GO:0070922 | RISC complex assembly | Biological process | 9 | 3 | 0.002 | 1 |
| GO:0010501 | RNA secondary structure unwinding | Biological process | 8 | 3 | 0.002 | 1 |
| GO:0021910 | smoothened signaling pathway involved in ventral spinal cord patterning | Biological process | 6 | 3 | 0.002 | 1 |
| GO:0046854 | phosphatidylinositol phosphate biosynthetic process | Biological process | 66 | 7 | 0.002 | 1 |
| GO:0002224 | toll-like receptor signaling pathway | Biological process | 111 | 9 | 0.002 | 1 |
| **KEGG pathway** | | | | | | |
| hsa04330 | Notch signaling pathway |  | 4 | 6 | 0.018 | 1 |
| hsa04630 | JAK-STAT signaling pathway |  | 111 | 8 | 0.020 | 1 |
| hsa00785 | Lipoic acid metabolism |  | 21 | 2 | 0.040 | 1 |
| hsa00562 | Inositol phosphate metabolism |  | 31 | 5 | 0.049 | 1 |
| hsa04620 | Toll-like receptor signaling pathway |  | 20 | 5 | 0.056 | 1 |
| hsa04071 | Sphingolipid signaling pathway |  | 32 | 7 | 0.061 | 1 |
| hsa03267 | Virion - Adenovirus |  | 27 | 1 | 0.062 | 1 |
| hsa04933 | AGE-RAGE signaling pathway in diabetic complications |  | 89 | 6 | 0.074 | 1 |
| hsa05217 | Basal cell carcinoma |  | 34 | 5 | 0.086 | 1 |
| hsa04930 | Type II diabetes mellitus |  | 220 | 4 | 0.100 | 1 |
| hsa00270 | Cysteine and methionine metabolism |  | 103 | 3 | 0.107 | 1 |
| hsa05017 | Spinocerebellar ataxia |  | 121 | 7 | 0.114 | 1 |
| hsa04371 | Apelin signaling pathway |  | 38 | 7 | 0.119 | 1 |
| hsa04062 | Chemokine signaling pathway |  | 209 | 8 | 0.120 | 1 |
| hsa05142 | Chagas disease |  | 31 | 5 | 0.122 | 1 |
| hsa00020 | Citrate cycle (TCA cycle) |  | 35 | 2 | 0.122 | 1 |
| hsa03082 | ATP-dependent chromatin remodeling |  | 35 | 5 | 0.129 | 1 |
| hsa00650 | Butanoate metabolism |  | 158 | 2 | 0.130 | 1 |
| hsa04340 | Hedgehog signaling pathway |  | 74 | 4 | 0.131 | 1 |
| hsa04360 | Axon guidance |  | 116 | 10 | 0.133 | 1 |

*FDR*, false discovery rate; *GO*, Gene Ontology; *KEGG*, Kyoto Encyclopedia of Genes and Genomes. Analysis was performed using the *missMethyl* R package, where the 589 CpGs identified from the EWAS meta-analysis were taken as the input and the 368,249 investigated CpGs were used as the background set of CpGs.

# Supplementary Table 10. Associations between the previously reported frailty-associated CpGs and the FI in the EWAS meta-analysis.

| **CpGs** | **Gene** | **Chr** | **Position** | **Study** | **PMID** | **Frailty measure** | **Reported direction of association** | **Meta-analysis in SATSA and LSADT samples** | | | | | |
| --- | --- | --- | --- | --- | --- | --- | --- | --- | --- | --- | --- | --- | --- |
|  |  |  |  |  |  |  |  | *β* (per 10% increase) | SE | *p* | *β* (frail vs. non-frail) | SE | *p* |
| cg01127300 | *-* | 22 | 38614796 | Gao et al., 2017, Epigenetics | 28001461 | FI | - | -0.005 | 0.003 | 0.073 | -0.014 | 0.008 | 0.064 |
| cg02657160 | *CPOX* | 3 | 98311063 | Gao et al., 2017, Epigenetics | 28001461 | FI | - | -0.001 | 0.001 | 0.260 | -0.002 | 0.002 | 0.327 |
| **cg05673882** | ***POLK*** | **5** | **74862702** | **Gao et al., 2017, Epigenetics** | **28001461** | **FI** | **-** | **-0.007** | **0.003** | **0.039** | **-0.023** | **0.008** | **0.007** |
| **cg07826859** | ***MYO1G*** | **7** | **45020086** | **Gao et al., 2017, Epigenetics** | **28001461** | **FI** | **-** | **-0.002** | **0.001** | **0.079** | **-0.005** | **0.003** | **0.048** |
| **cg14753356** | ***-*** | **6** | **30720108** | **Gao et al., 2017, Epigenetics** | **28001461** | **FI** | **-** | **-0.004** | **0.002** | **0.047** | **-0.013** | **0.005** | **0.009** |
| **cg19589396** | ***-*** | **8** | **103937374** | **Gao et al., 2017, Epigenetics** | **28001461** | **FI** | **-** | **-0.003** | **0.002** | **0.098** | **-0.013** | **0.006** | **0.023** |
| cg19859270 | *GPR15* | 3 | 98251294 | Gao et al., 2017, Epigenetics | 28001461 | FI | - | 0.000 | 0.001 | 0.510 | -0.001 | 0.002 | 0.662 |
| cg23667432 | *ALPP* | 2 | 233244439 | Gao et al., 2017, Epigenetics | 28001461 | FI | - | 0.000 | 0.001 | 0.765 | -0.005 | 0.003 | 0.131 |
| cg25189904 | *GNG12* | 1 | 68299493 | Gao et al., 2017, Epigenetics | 28001461 | FI | - | 0.003 | 0.004 | 0.478 | 0.006 | 0.010 | 0.516 |
| cg18314882 | *SHARPIN* | 8 | 145159734 | Gale et al., 2018, Clinical Epigenetics | 30075802 | FP | + | 0.000 | 0.000 | 0.821 | 0.000 | 0.000 | 0.875 |
| cg17588578 | *PCDHGA2* | 5 | 140723583 | Kim et al., 2018, GeroScience | 30136078 | FI | + | -0.002 | 0.004 | 0.696 | 0.001 | 0.012 | 0.942 |
| cg16421157 | *-* | 6 | 5036597 | Kim et al., 2018, GeroScience | 30136078 | FI | + | 0.004 | 0.004 | 0.370 | -0.002 | 0.010 | 0.819 |
| cg13072214 | *PARP10* | 8 | 145060799 | Kim et al., 2018, GeroScience | 30136078 | FI | - | -0.002 | 0.002 | 0.325 | -0.008 | 0.007 | 0.198 |
| cg15709766 | *APC2* | 19 | 1466497 | Kim et al., 2018, GeroScience | 30136078 | FI | + | -0.001 | 0.003 | 0.675 | 0.002 | 0.009 | 0.814 |
| cg00861009 | *HOXD11* | 2 | 176973688 | Kim et al., 2018, GeroScience | 30136078 | FI | + | 0.000 | 0.002 | 0.843 | 0.003 | 0.004 | 0.428 |
| **cg00291213** | ***RUNX1*** | **21** | **36398056** | **Li et al., 2022, Nature Communications** | **36071044** | **FI** | **-** | **-0.002** | **0.001** | **0.079** | **-0.011** | **0.003** | **0.002** |
| **cg08129092** | ***INTS3*** | **1** | **153746211** | **Li et al., 2022, Nature Communications** | **36071044** | **FI** | **-** | **-0.004** | **0.001** | **0.001** | **-0.007** | **0.003** | **0.013** |
| **cg05568549** | ***CCND3*** | **6** | **41907198** | **Li et al., 2022, Nature Communications** | **36071044** | **FI** | **-** | **-0.003** | **0.001** | **0.002** | **-0.009** | **0.003** | **0.001** |
| cg26542792 | *LOC283404* | 12 | 52603567 | Li et al., 2022, Nature Communications | 36071044 | FI | - | -0.001 | 0.001 | 0.577 | -0.004 | 0.003 | 0.254 |
| **cg06285727** | ***ATG16L2*** | **11** | **72524028** | **Li et al., 2022, Nature Communications** | **36071044** | **FI** | **-** | **-0.003** | **0.001** | **3.1E-04** | **-0.010** | **0.003** | **4.8E-04** |
| cg18065177 | *TLN2* | 15 | 63116127 | Li et al., 2022, Nature Communications | 36071044 | FI | - | -0.001 | 0.001 | 0.292 | 0.000 | 0.003 | 0.947 |
| **cg15687600** | ***-*** | **1** | **59280952** | **Li et al., 2022, Nature Communications** | **36071044** | **FI** | **-** | **-0.002** | **0.001** | **0.003** | **-0.006** | **0.002** | **0.004** |
| cg03147185 | *NCAPH* | 2 | 97008030 | Li et al., 2022, Nature Communications | 36071044 | FI | - | -0.003 | 0.002 | 0.099 | -0.007 | 0.005 | 0.140 |
| **cg25607249** | ***SLC1A5*** | **19** | **47288039** | **Li et al., 2022, Nature Communications** | **36071044** | **FI** | **-** | **-0.003** | **0.001** | **0.002** | **-0.007** | **0.003** | **0.011** |
| **cg00252813** | ***GAPDH*** | **12** | **6642229** | **Li et al., 2022, Nature Communications** | **36071044** | **FI** | **-** | **-0.004** | **0.001** | **0.001** | **-0.007** | **0.003** | **0.011** |
| **cg23190089** | ***SLC22A18AS*** | **11** | **2920209** | **Li et al., 2022, Nature Communications** | **36071044** | **FI** | **-** | **-0.004** | **0.001** | **1.2E-04** | **-0.009** | **0.002** | **2.7E-04** |
| **cg22799757** | ***SCRN1*** | **7** | **29997642** | **Li et al., 2022, Nature Communications** | **36071044** | **FI** | **-** | **-0.003** | **0.001** | **0.015** | **-0.006** | **0.003** | **0.071** |
| **cg23458887** | ***-*** | **10** | **36569926** | **Li et al., 2022, Nature Communications** | **36071044** | **FI** | **-** | **-0.003** | **0.001** | **0.025** | **-0.007** | **0.004** | **0.064** |
| cg15058210 | *HDAC4* | 2 | 240196877 | Li et al., 2022, Nature Communications | 36071044 | FI | - | -0.003 | 0.002 | 0.156 | -0.006 | 0.005 | 0.209 |
| **cg21766592** | ***SLC1A5*** | **19** | **47288066** | **Li et al., 2022, Nature Communications** | **36071044** | **FI** | **-** | **-0.004** | **0.001** | **0.001** | **-0.010** | **0.003** | **0.002** |
| **cg06811800** | ***ATP4B*** | **13** | **114312105** | **Li et al., 2022, Nature Communications** | **36071044** | **FI** | **-** | **-0.002** | **0.001** | **0.034** | **-0.008** | **0.003** | **0.007** |
| cg19267254 | *CASP9* | 1 | 15828075 | Li et al., 2022, Nature Communications | 36071044 | FI | - | 0.000 | 0.002 | 0.817 | -0.007 | 0.006 | 0.194 |
| **cg12510708** | ***NFE2L3*** | **7** | **26193805** | **Li et al., 2022, Nature Communications** | **36071044** | **FI** | **-** | **-0.004** | **0.002** | **0.010** | **-0.013** | **0.005** | **0.005** |
| **cg15380836** | ***RILP*** | **17** | **1553341** | **Li et al., 2022, Nature Communications** | **36071044** | **FI** | **-** | **-0.002** | **0.001** | **0.063** | **-0.005** | **0.002** | **0.040** |
| **cg01406381** | ***SLC1A5*** | **19** | **47288263** | **Li et al., 2022, Nature Communications** | **36071044** | **FI** | **-** | **-0.003** | **0.001** | **0.002** | **-0.005** | **0.002** | **0.033** |
| cg11700584 | *RPL36AL* | 14 | 50088544 | Li et al., 2022, Nature Communications | 36071044 | FI | - | -0.003 | 0.002 | 0.150 | -0.002 | 0.006 | 0.723 |
| **cg01234420** | ***LOC150381*** | **22** | **46453808** | **Li et al., 2022, Nature Communications** | **36071044** | **FI** | **-** | **-0.004** | **0.002** | **0.046** | **-0.011** | **0.006** | **0.038** |
| cg00377681 | *-* | 6 | 37102603 | Li et al., 2022, Nature Communications | 36071044 | FI | - | 0.001 | 0.001 | 0.621 | -0.001 | 0.004 | 0.867 |
| **cg22933646** | ***EEF1G*** | **11** | **62342512** | **Li et al., 2022, Nature Communications** | **36071044** | **FI** | **-** | **-0.002** | **0.001** | **0.043** | **-0.005** | **0.002** | **0.027** |
| cg17860366 | *ABHD2* | 15 | 89634741 | Li et al., 2022, Nature Communications | 36071044 | FI | - | N/A | N/A | N/A | N/A | N/A | N/A |
| cg19556613 | *-* | 5 | 116280275 | Li et al., 2022, Nature Communications | 36071044 | FI | - | -0.001 | 0.001 | 0.497 | -0.003 | 0.003 | 0.290 |
| cg21656937 | *-* | 12 | 54086662 | Li et al., 2022, Nature Communications | 36071044 | FI | - | -0.004 | 0.003 | 0.301 | -0.011 | 0.010 | 0.258 |

**Supplementary Table 10.** (*continued*)

| **CpGs** | **Gene** | **Chr** | **Position** | **Study** | **PMID** | **Frailty measure** | **Reported direction of association** | **Meta-analysis in SATSA and LSADT samples** | | | | | |
| --- | --- | --- | --- | --- | --- | --- | --- | --- | --- | --- | --- | --- | --- |
|  |  |  |  |  |  |  |  | *β* (per 10% increase) | SE | *p* | *β* (frail vs. non-frail) | SE | *p* |
| **cg03725309** | ***SARS*** | **1** | **109757585** | **Li et al., 2022, Nature Communications** | **36071044** | **FI** | **-** | **-0.003** | **0.001** | **0.011** | **-0.005** | **0.003** | **0.156** |
| **cg05809481** | ***ISG20L2*** | **1** | **156696467** | **Li et al., 2022, Nature Communications** | **36071044** | **FI** | **-** | **-0.004** | **0.002** | **0.011** | **-0.012** | **0.005** | **0.010** |
| cg13119578 | *-* | 11 | 65196696 | Li et al., 2022, Nature Communications | 36071044 | FI | - | N/A | N/A | N/A | N/A | N/A | N/A |
| cg01340312 | *TUBB* | 6 | 30687511 | Li et al., 2022, Nature Communications | 36071044 | FI | - | -0.001 | 0.001 | 0.260 | -0.003 | 0.003 | 0.251 |
| cg02556005 | *-* | 16 | 74310568 | Li et al., 2022, Nature Communications | 36071044 | FI | - | -0.001 | 0.001 | 0.630 | -0.002 | 0.003 | 0.622 |
| **cg02519286** | ***GAPDH*** | **12** | **6642354** | **Li et al., 2022, Nature Communications** | **36071044** | **FI** | **-** | **-0.003** | **0.001** | **0.008** | **-0.006** | **0.003** | **0.017** |
| cg17345741 | *-* | 14 | 69472652 | Li et al., 2022, Nature Communications | 36071044 | FI | - | -0.001 | 0.001 | 0.282 | -0.002 | 0.003 | 0.479 |
| **cg17971578** | ***STK40*** | **1** | **36852463** | **Li et al., 2022, Nature Communications** | **36071044** | **FI** | **-** | **-0.003** | **0.001** | **0.021** | **-0.011** | **0.004** | **0.005** |
| **cg26954174** | ***NOD2*** | **16** | **50730813** | **Li et al., 2022, Nature Communications** | **36071044** | **FI** | **-** | **-0.004** | **0.001** | **0.002** | **-0.010** | **0.004** | **0.008** |
| cg18791730 | *HAO2* | 1 | 119910205 | Li et al., 2022, Nature Communications | 36071044 | FI | - | -0.002 | 0.002 | 0.213 | -0.005 | 0.005 | 0.335 |
| cg27115863 | *-* | 22 | 37921640 | Li et al., 2022, Nature Communications | 36071044 | FI | - | -0.003 | 0.002 | 0.176 | -0.008 | 0.006 | 0.182 |
| cg04955914 | *C2orf24* | 2 | 220040571 | Li et al., 2022, Nature Communications | 36071044 | FI | - | -0.001 | 0.002 | 0.398 | -0.006 | 0.005 | 0.229 |
| **cg08354751** | ***-*** | **16** | **11450712** | **Li et al., 2022, Nature Communications** | **36071044** | **FI** | **-** | **-0.003** | **0.001** | **0.037** | **-0.011** | **0.003** | **0.001** |
| cg00921350 | *SNX20* | 16 | 50701499 | Li et al., 2022, Nature Communications | 36071044 | FI | - | -0.002 | 0.002 | 0.178 | -0.006 | 0.005 | 0.192 |
| **cg11993160** | ***-*** | **18** | **12404041** | **Li et al., 2022, Nature Communications** | **36071044** | **FI** | **-** | **-0.005** | **0.002** | **0.017** | **-0.010** | **0.005** | **0.049** |
| cg00640314 | *SNORD87* | 8 | 67835853 | Li et al., 2022, Nature Communications | 36071044 | FI | - | N/A | N/A | N/A | N/A | N/A | N/A |
| cg07312601 | *MRTO4* | 1 | 19583887 | Li et al., 2022, Nature Communications | 36071044 | FI | - | -0.003 | 0.002 | 0.180 | -0.010 | 0.006 | 0.101 |
| **cg04742397** | ***-*** | **14** | **95986554** | **Li et al., 2022, Nature Communications** | **36071044** | **FI** | **-** | **-0.004** | **0.002** | **0.013** | **-0.011** | **0.004** | **0.010** |
| cg10424974 | *-* | 16 | 66388348 | Li et al., 2022, Nature Communications | 36071044 | FI | - | -0.003 | 0.002 | 0.222 | -0.009 | 0.005 | 0.097 |
| **cg07092212** | ***DGKZ*** | **11** | **46382544** | **Li et al., 2022, Nature Communications** | **36071044** | **FI** | **-** | **-0.003** | **0.001** | **0.030** | **-0.005** | **0.003** | **0.083** |
| **cg24046474** | ***RPL12*** | **9** | **130212550** | **Li et al., 2022, Nature Communications** | **36071044** | **FI** | **-** | **-0.003** | **0.002** | **0.051** | **-0.009** | **0.004** | **0.024** |
| cg01491071 | *-* | 2 | 206789816 | Li et al., 2022, Nature Communications | 36071044 | FI | - | 0.004 | 0.003 | 0.133 | 0.011 | 0.008 | 0.202 |
| cg08998192 | *TAP2* | 6 | 32805570 | Li et al., 2022, Nature Communications | 36071044 | FI | - | -0.003 | 0.002 | 0.247 | -0.007 | 0.007 | 0.296 |
| **cg04772644** | ***RXRB*** | **6** | **33165428** | **Li et al., 2022, Nature Communications** | **36071044** | **FI** | **-** | **-0.004** | **0.001** | **0.001** | **-0.011** | **0.003** | **4.7E-04** |
| **cg02867102** | ***-*** | **17** | **62398693** | **Li et al., 2022, Nature Communications** | **36071044** | **FI** | **-** | **-0.003** | **0.001** | **0.049** | **-0.009** | **0.004** | **0.016** |
| cg08463758 | *EMILIN3* | 20 | 39995649 | Li et al., 2022, Nature Communications | 36071044 | FI | + | -0.001 | 0.002 | 0.715 | 0.002 | 0.006 | 0.733 |
| cg11084334 | *LHFPL4* | 3 | 9594264 | Li et al., 2022, Nature Communications | 36071044 | FI | + | -0.002 | 0.002 | 0.174 | -0.006 | 0.005 | 0.222 |
| cg13570972 | *PAX6* | 11 | 31839632 | Li et al., 2022, Nature Communications | 36071044 | FI | + | 0.001 | 0.002 | 0.527 | 0.001 | 0.005 | 0.862 |
| **cg26126740** | ***TOX2*** | **20** | **42574332** | **Li et al., 2022, Nature Communications** | **36071044** | **FI** | **+** | **0.002** | **0.001** | **0.033** | **0.004** | **0.002** | **0.031** |
| cg15135047 | *-* | 3 | 72149514 | Li et al., 2022, Nature Communications | 36071044 | FI | + | -0.001 | 0.001 | 0.364 | -0.003 | 0.003 | 0.188 |
| cg00259097 | *VAC14* | 16 | 70770604 | Li et al., 2022, Nature Communications | 36071044 | FI | + | 0.002 | 0.001 | 0.097 | 0.004 | 0.003 | 0.174 |
| cg07349348 | *-* | 8 | 142253047 | Li et al., 2022, Nature Communications | 36071044 | FI | + | -0.001 | 0.001 | 0.382 | -0.002 | 0.002 | 0.274 |
| cg10408430 | *P4HA3* | 11 | 73979900 | Li et al., 2022, Nature Communications | 36071044 | FI | + | -0.001 | 0.001 | 0.098 | -0.003 | 0.002 | 0.172 |
| cg16125214 | *PIK3CD* | 1 | 9751353 | Li et al., 2022, Nature Communications | 36071044 | FI | + | 0.000 | 0.001 | 0.631 | 0.001 | 0.002 | 0.538 |
| cg17220749 | *GALNT2* | 1 | 230249292 | Li et al., 2022, Nature Communications | 36071044 | FI | + | 0.000 | 0.001 | 0.673 | -0.001 | 0.002 | 0.791 |
| cg04550823 | *LOC339524* | 1 | 87617916 | Li et al., 2022, Nature Communications | 36071044 | FI | + | 0.000 | 0.001 | 0.890 | -0.001 | 0.003 | 0.608 |
| cg08469255 | *DDR1* | 6 | 30851069 | Li et al., 2022, Nature Communications | 36071044 | FI | + | 0.001 | 0.001 | 0.527 | 0.003 | 0.003 | 0.357 |
| cg08944236 | *CHD9* | 16 | 53242355 | Li et al., 2022, Nature Communications | 36071044 | FI | + | -0.004 | 0.001 | 0.003 | -0.012 | 0.004 | 0.002 |

*Chr*, chromosome; *FDR*, false discovery rate; *FI*, frailty index; *FP*, frailty phenotype; *LSADT*, Longitudinal Study of Aging Danish Twins; *SATSA*, Swedish Adoption/Twin Study of Aging; *SE; standard error*. The 34 bolded CpGs were associated with either the continuous FI or categorical FI at *p*<.05 in the EWAS meta-analysis and directionally consistent with the previously reported association. Genome position was based on genome assembly GRCh37 (hg19).
